# Antecedents, Consequences, and the Role of Third Parties in the Trust Repair Process: Evidence Taken from Orthodontics

**DOI:** 10.3390/healthcare10101811

**Published:** 2022-09-20

**Authors:** Jyh-Jeng Wu, Paul C. Talley, Kuang-Ming Kuo, Jia-Lin Chen

**Affiliations:** 1 Department of Business Management, National United University, Miaoli 360301, Taiwan; 2 Department of Applied English, I-Shou University, Kaohsiung City 84001, Taiwan

**Keywords:** medical disputes, satisfaction, third-party trust repair, trust repair strategy, word-of-mouth behavior, mediation

## Abstract

Orthodontic treatment has popularized in Taiwan. Healthcare institutions can be responsive in their coping strategies and determine whether third-party intervention should take place involving medical disputes related to orthodontics in order to repair patient trust. This study draws on orthodontic treatment to explore the effect of various trust repair strategies employed by healthcare institutions and third-party involvement positively affecting outcomes related to trust repair. Patients were recruited among those who have undergone orthodontic treatments, and 353 valid scenario-based questionnaires were collected through an online survey. Results revealed that: (1) the affective and informational repair strategies positively impacted trust repair while the functional repair strategy did not; (2) trust repair positively impacted patient satisfaction/word-of-mouth and mediated between repair strategies and satisfaction/word-of-mouth; and (3) third-party involvement moderated the relationship between trust repair and word-of-mouth. The findings suggest that rather than receiving monetary compensation, patients usually prefer that healthcare institutions acknowledge their fault, offer apologies, and engage in active communications to clarify the causes of medical dispute. Further, an objective third party should be involved to mediate the medical disputes to afford satisfaction all around.

## 1. Introduction

Patients must be able to trust in their healthcare providers. According to the Ministry of Health and Welfare of Taiwan statistics [1], Taiwan recorded a total of 12,144 cases of litigation involving medical disputes between 1987 and 2021. Of these, 992 were confirmed cases of medical negligence on the part of a physician, accounting for around 8% of all reviewed cases of medical disputes. Taken together with the 604 reviewed cases of potential negligence, they represented 13% of all reviewed cases of medical disputes. In other words, roughly 85% of the physicians involved in these disputes had to shoulder the burden of medical lawsuits without having committed any demonstrable medical negligence.

Trust is an essential factor in healthcare processes because patient trust inevitably leads to improved doctor–patient relationships that foster confidence in healthcare professionals [2,3]. The prediction of the outcome has been referred to as a possible instrument for patient communication [4]. In the event of a medical dispute in which a patient’s original trust is potentially compromised, trust repair retains a vital role. Previous trust-related literature has proposed a variety of strategies for the repair of broken trust variables. For instance, Mazor, et al. [5] suggested that information disclosure may reduce claims associated with medical accidents; Hobgood, et al. [6] suggested the disclosure of errors as a means to reduce anger and litigation because policies of full disclosure, apology, and compensation can lower the cost of litigation [7]. These are all strategies that can reduce finger-pointing and anger among members of the public while positively affecting trust [8]. Another common approach is to offer victims financial compensation because economic exchange relationships are considered to be driven by tangible outcomes [9]. Bottom, et al. [10] attested that cooperation can be effectively rebuilt when substantial financial compensation is offered by the transgressor to the perceived victim(s).

With the advancement of modernity and growing socio-economic prosperity, many people are paying increasing attention to the effects of one’s dentition on aesthetic judgments and social activities [11,12]. In recent years, there has been a burgeoning growth in the orthodontic industry and a widespread desire for clean, white, and straight teeth. Presumed dental beauty is a vital ingredient in a well-groomed appearance, with a number of studies indicating that orthodontic treatments can bring socio-psychological benefits such as improved aesthetics and reduced social anxiety [13,14]. Owing to the widespread popularity of corrective dental healthcare, growing susceptibility to medical disputes and the long-term need for follow-up appointments mean that orthodontic treatments must repeatedly confront the problem of repairing patients’ trust. Thus, the ability to repair patients’ trust in the event of a dispute has become an indispensable management skill for healthcare institutions to attain; even so, insufficient theoretical and scholarly attention has been afforded to the effective restoration of broken trust by these institutions [15,16]. Moreover, prior studies in this regard have focused largely on the compensatory strategies adopted by healthcare institutions for the victims per se [17] and rarely touched on the effects of involving an independent, and presumably impartial, third party in medical disputes involving healthcare providers and patients.

To elucidate the above research topics, the current study aims to do the following: (1) it will explore the effects of different trust repair strategies (i.e., affective, functional, and informational) on trust repair efforts; (2) it will discuss the effects on the outcomes of trust repair (i.e., satisfaction and word-of-mouth); (3) it will examine whether third-party involvement has an evident effect on the relationship between trust repair and its outcomes; and (4) it will investigate whether trust repair plays a mediating role between trust repair strategies and the outcomes of trust repair.

## 2. Conceptual Development 

### 2.1. Medical Dispute and Patient Trust Repair

Trust is a psychological state comprising intentions to expose one’s vulnerability to others and expectations about others’ intentions to carry out good actions in the future [18]. It is crucial to the smooth functioning of a healthcare system [19]. Trust in healthcare is often referred to as the patient’s belief that the physician will beneficially care for their interests [20]. In the event of a medical dispute, patients may promulgate an argument related to a perceived medical error due to an alleged claim that a physician’s failure to fulfill his/her practical care obligations has exacerbated the patient’s pre-existing condition [21]. This breakdown in the trust environment tends to create tension between both the patient and the physician or the healthcare institution. More specifically, it damages the former’s trust in the physician and the healthcare institution overall [22]. As a result, healthcare institutions have endeavored to keep the occurrence of litigious or onerous medical disputes to a minimum, but the number continues to climb every year [23].

**Figure 1:**
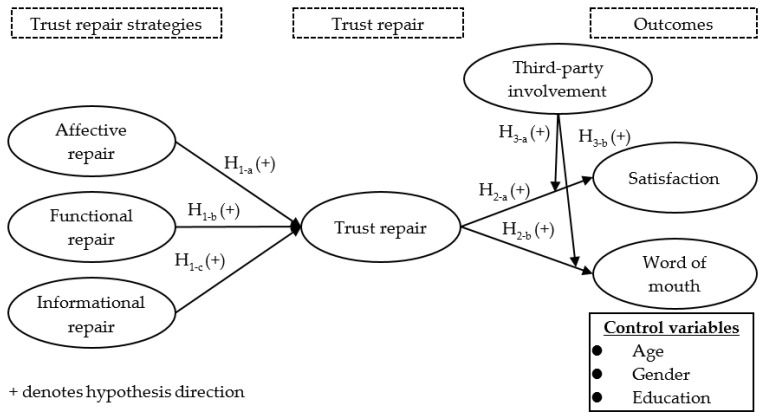
Research framework for antecedents, consequences, and the role of third parties in the trust repair process.

Trust remains important in the interactions between patients and healthcare institutions [24,25]. Hence, after a medical dispute has precipitated, the healthcare institution adopts corresponding trust repair strategies in an attempt to restore patients’ trust and to avoid negative repercussions for the reputation and even on-going operations of the healthcare institution. Thus, determining which trust repair strategies should be adopted and whether or not third-party involvement is conducive to trust repair are issues in need of further clarification. Thus, the main purpose of this study is to identify any significant correlation between trust repair and the various trust repair strategies (i.e., affective, functional, and informational repair) undertaken by healthcare institutions after the occurrence of a potential medical dispute. The current study would also like to suggest that trust repair serves as a mediator between all three types of trust repair strategies and satisfaction and word-of-mouth, whereas third-party involvement exerts a moderating effect that can enhance the relationships between trust repair and satisfaction and word-of-mouth. To diminish the impact of the confounding variables, age, gender, and level of education were included as control variables. The research framework of this study is illustrated in Figure 1.

### 2.2. Trust Repair Strategies

Given the significance of organizational trust repair efforts, trust repair theory has been widely discussed in academic studies, with the strategies of repair central to their focus [26,27]. A variety of trust repair strategies have been proposed [26], but according to Xie and Peng [28], they can be categorized into three main types: affective, functional, and informational. In the case of the healthcare industry, when a medical dispute arises from a perceived failure or error in the medical services provided, the healthcare institution or physician will adopt repair strategies to relieve tension with the patient and improve satisfaction.

#### 2.2.1. Affective Repair

Affective repair represents the affective means of compensating the patient through specific behaviors such as expressing genuine care for the patient, sincere apologizing, and a heartfelt showing of remorse. Kim, et al. [29] defined an apology as a statement that acknowledges responsibility and regret for a violation of trust. When a transgression takes place, an apology is a typically affective approach to repair interpersonal trust or the trust between individuals and an organization [29,30]. Tomlinson, et al. [31] found that an apology can increase a perceived victim’s willingness to reconcile, while Harrison-Walker [32] demonstrated that consumers are more willing to negotiate with firms that demonstrate the ability to apologize. In healthcare scenarios, affective expressions such as a display of kindness, active listening, proper eye contact, and smiling expressions can enhance patients’ trust towards a physician [33]. Other studies discovered that patients will develop a meaningful sense of trust toward physicians who listen to and care for them [34]. In light of the above literature, this study hypothesized the following:

**Hypothesis** **1** **(H1-a).** *Affective repair strategies adopted by healthcare institutions have a positive effect on trust repair.*

#### 2.2.2. Functional Repair

Functional repair is the act of offering a patient substantive compensation by means of monetary refunds or discounts and promotions. Compensating customers is a common strategy for service recovery that can help dissipate consumers’ anger and discontent after service failures [35]. Its effectiveness in repairing trust increases with the relative compensation amount, and it may be amplified when adopted in combination with apologies [35,36]. In the context of this study, where a medical dispute arises after a patient undergoes an orthodontic treatment, monetary compensation offered by the respective healthcare institution should be ample to restore the patient’s trust. According to the above literature and discussions, this study hypothesized the following:

**Hypothesis** **1** **(H1-b).** *Functional repair strategies adopted by healthcare institutions have a positive effect on trust repair.*

#### 2.2.3. Informational Repair

Informational repair occurs when a healthcare institution explains and clarifies information for patients and then adopts suitable measures to communicate with them. When dealing with a crisis, organizations that showcase evidence, clarify facts, and disclose the latest information do so in the hope of resolving any misunderstanding caused by insufficient information via informational repair [37,38]. Chen, et al. [39] have pointed out that informational repair cannot only satisfy consumers’ expectations, but it can also attenuate the tension between consumers and firms and its negative outcomes. Guo, et al. [40] found that the abundance of information controlled by consumers positively correlated with their satisfaction with an organization’s crisis recovery efforts. Based on the above literature and discussions, this study hypothesized the following:

**Hypothesis** **1** **(H1-c).** *Informational repair strategies adopted by healthcare institutions have a positive effect on trust repair.*

### 2.3. Outcomes of Trust Repair

Trust is regarded as a crucial antecedent for consumer behavior, loyalty, or customer-based brand equity [41,42]. When there is a problem with the services offered by an organization, consumers’ trust in the firm will be compromised, requiring the organization to retain its consumers through efforts such as service recovery or trust repair [43]. A prior meta-analysis [44] identified the outcomes of service recovery as satisfaction, re-purchase intention, word-of-mouth, and corporate image. Trust repair, broadly speaking, shares a similar purpose with service recovery, in such a manner that their potential outcomes should thus be similar. The personally time-consuming and costly nature of orthodontic treatment means that the re-purchase intention for most people remains rather limited. The image of healthcare institutions, while important, is inherently different from that of other organizations, since most patients do not have sufficient specialized knowledge to assess the complex services rendered by the healthcare institutions [45], which may also further complicate how patients perceive the image of healthcare institutions. Thus, this study adopted satisfaction and word-of-mouth only as the eventual outcomes of trust repair.

#### 2.3.1. Satisfaction

In the context of healthcare, satisfaction refers to patients’ overall evaluation after receiving healthcare services [46], comprising appraisals of the efficacy, safety, and benefits of the healthcare services provided [47]. Patient satisfaction is also a subject of increasing importance among healthcare institutions’ planning [48]. Patients are more likely to be satisfied if the staff members of a healthcare institution are competent, approachable, kind, courteous, and friendly [49]. In addition, satisfied patients tend to seek medical attention repeatedly at the same healthcare institution if they have additional healthcare needs, and then they are more likely to recommend that healthcare institution to others [50]. A previous study found that consumers’ satisfaction increases with their trust in an organization [51]. The same applies to healthcare contexts: Hillen, et al. [52] proved that patients’ satisfaction increases with a trust in their physician. In light of the above literature and discussions, this study hypothesized the following:

**Hypothesis** **2** **(H2-a).** *Trust repair by healthcare institutions has a positive effect on satisfaction.*

#### 2.3.2. Word-of-Mouth

Word-of-mouth is always considered to have a significant influence on consumers’ attitudes and decision-making processes [53]. Consumers who have established a relationship with a trusted brand are likely to spread positive word-of-mouth about that brand [54]. Consumers may purchase something on the basis of word-of-mouth recommendations from family members or relatives [55]. Trust may even influence customer retention and word-of-mouth communication [56]. Moreover, organizations that reinforce their commitments to customers may gain more positive word-of-mouth [57]. In healthcare contexts, patients with medical needs tend to actively seek specific healthcare information [58]. In addition, family members and relatives may ask patients who have experienced a particular healthcare service for advice [59]. Public trust also exerts a positive influence on word-of-mouth [60,61]. Considering the above literature and discussion, this study hypothesized the following:

**Hypothesis** **2** **(H2-b).** *Trust repair by healthcare institutions has a positive effect on word-of-mouth.*

### 2.4. The Moderating Role of Third-Party Involvement

In the event of a medical dispute, the healthcare institution may choose to initiate negotiations with the patient, offering him/her a variety of compensatory measures. This may possibly involve an impartial third party to mediate the dispute in order to help narrow the cognitive gap between the two parties, resolve the dispute, and facilitate reconciliation. Previous literature has shed light on the importance of third-party involvement to achieve trust building [62] and trust repair [63]. Third parties can act as mediators who relieve the tension between the two disputants by persuasion [64]. Kolb [65] suggested that third parties indirectly facilitate reconciliation by conversely satisfying the needs of the victim and the alleged perpetrator. In other words, when there is a dispute between an organization and its consumer, third-party involvement in mediation can improve the subsequent outcomes of repair. Within the context of this study, if a third party can mediate a medical dispute between a patient undergoing orthodontic treatment and a healthcare institution, and in restoring the former’s trust in the latter, the patient may become satisfied with that healthcare institution and may prove willing to spread positive word-of-mouth commentary about its services. Hence, this study proposed the following hypotheses:

**Hypothesis** **3** **(H3-a).** *Third-party involvement can enhance the relationship between trust repair and patient satisfaction.*

**Hypothesis** **3** **(H3-b).** *Third-party involvement can enhance the relationship between trust repair and patients’ word-of-mouth.*

### 2.5. The Mediating Role of Trust Repair

To organizations, consumer trust is a valuable strategic asset that must be protected persistently [66]. Any perceived violation of trust by an organization can jeopardize its relationship with customers, and it can even strip it of its competitive advantages [66,67], thus demonstrating the importance of trust to organizations. As a result, organizations experiencing broken customer trust must conduct trust repair in two phases [31]. The first phase involves a willingness-to-reconcile, where reconciliation is considered as a behavioral manifestation of forgiveness, implying a willingness-to-accept the trust repair strategies proposed by the organization. This may be performed in-line with restoring the customers’ trust. The second phase involves the intention to continue cooperation, which indicates that the customers are satisfied with the organization’s services, and they may even engage in word-of-mouth communication [44]. With regards to the context of this study, the main purpose of the trust repair strategies proposed by healthcare institutions in response to medical disputes (i.e., affective, functional, and informational) are to affect trust repair, which is a prerequisite of patient satisfaction and willingness-to-partake in word-of-mouth communication. To put it differently, trust repair should play a mediating role between trust repair strategies and patient satisfaction and word-of-mouth. Thus, this study proposed the following hypotheses:

**Hypothesis** **4** **(H4-a).** *Trust repair plays a mediating role between affective repair and patient satisfaction.*

**Hypothesis** **4** **(H4-b).** *Trust repair plays a mediating role between functional repair and patient satisfaction.*

**Hypothesis** **4** **(H4-c).** *Trust repair plays a mediating role between informational repair and patient satisfaction.*

**Hypothesis** **4** **(H4-d).** *Trust repair plays a mediating role between affective repair and patients’ word-of-mouth.*

**Hypothesis** **4** **(H4-e).** *Trust repair plays a mediating role between functional repair and patients’ word-of-mouth.*

**Hypothesis** **4** **(H4-f).** *Trust repair plays a mediating role between informational repair and patients’ word-of-mouth.*

## 3. Research Method

### 3.1. Measures

The scale items that were developed based on validated instruments are taken from prior relevant literature and adapted to the research context. The first draft of the questionnaire was reviewed by three experts (including two dentists and a professor in marketing) and modified based on their recommendations. With reference to Xie and Peng [28], affective, functional, and informational repair strategies were each measured using three items. Trust repair was assessed using four items mainly based on the study by Sedikides [68]. Satisfaction was evaluated using three items mainly adapted from the previous literature [69,70,71]. Word-of-mouth was measured using three items adapted from Babin, et al. [72]. All of the questionnaire items were rated on a five-point Likert scale, with 1 representing “extremely disagree” and 5 indicating “extremely agree.” Prior to the official distribution of the questionnaire, a pre-test was undertaken with 86 university students aged between 18 and 22, and the statements used in the questionnaire items were slightly fine-tuned based on the pre-test results. The final questionnaire is shown in Appendix A.

### 3.2. Data Collection and Sample

We conducted a convenience sampling of Taiwanese residents via an online survey. An invitation message including the purpose-of-study, the sampling procedure, and protection of participants’ privacy and anonymity, and a URL link to the questionnaire was posted on orthodontic-related groups on Facebook between 17 January and 17 February 2022. Individuals who had experience with prior or on-going orthodontic treatment were qualified to participate in this survey. As the purpose of this study was to explore the trust repair undertaken by healthcare institutions after the occurrence of medical disputes, a hypothetical scenario-based method with two scenarios was adopted. The first involved trust repair undertaken by the healthcare institutions alone, while the second involved an additional third party involved in the tentative mediation process. When filling out the questionnaire online, the subjects were randomly assigned one of these scenarios on which to base their answers (see Appendix B). IP addresses were used to ensure that each subject could only complete the questionnaire once. Our study protocol qualified exemption of Institutional Review Board review because of its anonymity, non-interaction, non-intrusion, and conduct in a public setting, and no specific individual was identifiable from the information collected [73].

### 3.3. Statistical Analysis

The collected data were first analyzed using descriptive statistics to understand the distribution of sample characteristics. Afterward, hypothesis testing was carried out by means of structural equation modeling. The moderating functions of third-party involvement were tested using the steps suggested by Baron and Kenny [74], while the mediation effects were verified using the method suggested by MacKinnon, et al. [75]. The significance level was set at 0.05 across the study.

## 4. Results

### 4.1. Descriptive Statistics

This study retrieved 362 questionnaires, of which 353 were valid, and 9 excluded for failure to meet the inclusion criteria. The sample consisted mainly of females (66.57%) and individuals in the age group of 21–30 years (41.64%). Most of the subjects reported an educational level of college/university (76.77%), while having previously spent between USD 1700 and USD 2650 on their orthodontic treatment (45.89%). There were 147 responses (41.64%) obtained for the first scenario (i.e., without third-party involvement) and 206 (58.36%) for the second scenario (i.e., with third-party involvement). The respondents’ demographic information is summarized in Table 1.

### 4.2. Measurement Model

Structural equation modeling was performed using the two-step approach suggested by Anderson and Gerbing [76], which involved a measurement model and a structural model. The measurement model, otherwise known as a confirmatory factor analysis, mainly tests for the reliability and validity of measurable variables and latent variables. In terms of reliability, as shown in Table 2, the measurable variables obtained in this study all yielded a factor loading above the recommended value of 0.5 [77], and the latent variables all reported a composite reliability score exceeding the recommended value of 0.7 [77]. In terms of validity, Table 2 shows that the average variance extracted (AVE) for each of the latent variables exceeded the recommended value of 0.5, thereby indicating that the latent variables had satisfactory convergent validity [78]. Discriminant validity was evaluated using two different criteria: one proposed by Fornell and Larcker [78] and the other by Henseler, et al. [79]. As shown in Table 3, the square root of the AVE for each of the latent variables was greater than the correlation coefficients between these variables, fulfilling the criterion suggested by Fornell and Larcker [78]. The heterotrait–monotrait ratios of the correlations between the latent variables were also consistently below the recommended threshold of 0.85 [79], demonstrating satisfactory discriminant validity (as shown in Table 3).

### 4.3. Structural Model

According to the results of the structural model analysis, affective repair was found to significantly and positively predict trust repair (β = 0.27, *p* < 0.001), supporting H1-a. However, H1-b was rejected since functional repair did not predict trust repair (β = 0.00, *p* = 0.989). Since informational repair also significantly and positively predicted trust repair (β = 0.59, *p* < 0.001), H1-c was validated. Trust repair was found to significantly and positively predict satisfaction (β = 0.82, *p* < 0.001) and word-of-mouth (β = 0.76, *p* < 0.001), confirming both H2-a and H2-b. The results of the hypothesis testing are outlined in Table 4. In terms of the explanatory power of the model, affective, functional, and informational repair explained about 63.76% of the variance in trust repair altogether, whereas trust repair explained roughly 67.84% and 57.27% of the variance in satisfaction and word-of-mouth, respectively. With regard to the control variables, whether the model included age, gender, and education had no effect on the final outcomes of hypothesis testing, indicating that the potential influence of these three variables had been controlled for.

Because the data analysis was conducted via structural equation modeling in this study, additional evaluations of the model fit were required. As shown in Table 5, both the measurement model and the structural model were able to meet all of the recommended thresholds across the absolute, incremental, and parsimonious fit indices [77,80,81,82], signifying the reasonably good fit of the models in this study.

### 4.4. Moderating Effect of Third-Party Involvement

To understand whether third-party involvement plays a moderating role between trust repair and satisfaction/word-of-mouth, this study divided the respondents into two groups, one without third-party involvement (*n* = 147) and the other with (*n* = 206), based on the scenario to which they responded during questionnaire completion. A multi-group analysis [83,84] was then employed to compare the path coefficients derived from different sub-samples. To begin with, the omnibus Wald test yielded a result of χ^2^(2, 353) = 6.03, *p* = 0.019, which established third-party involvement as a significant moderator variable. Further testing of the inter-group differences in individual paths also revealed that the path coefficient between trust repair and patient satisfaction was higher in the sample group with third-party involvement than in the one without, but the difference failed to achieve statistical significance (0.86 vs. 0.76, *p* = 0.854); hence, H3-a was rejected. The path coefficient between trust repair and word-of-mouth was significantly higher in the group with third-party involvement than in the one without (0.81 vs. 0.67, *p* = 0.019); thus, H3-b was supported (as shown in Table 6).

### 4.5. Mediating Effect of Trust Repair

To examine the mediating effect of trust repair, this study applied the product distribution proposed by MacKinnon, Lockwood, Hoffman, West and Sheets [75] to estimate the confidence intervals and, in turn, determine whether there was any mediating effect. Since Table 7 shows that 95% confidence intervals did not include zero and that affective repair could directly predict patient satisfaction, trust repair was found to partially mediate the relationship between affective repair and satisfaction; hence, H4-a was validated. By extension, using the same form of logic, trust repair was also determined to partially mediate the relationship between functional repair and satisfaction and that found between informational repair and satisfaction, thus supporting H4-b and H4-c, respectively. Furthermore, trust repair partially mediated the relationship between affective repair and word-of-mouth, but it exhibited no mediation effect between functional repair and word-of-mouth, validating H4-d but rejecting H4-e. Trust repair was also revealed to fully mediate the relationship between informational repair and word-of-mouth, confirming H4-f.

## 5. Discussion

When discussing consumers’ negative reactions to failed transactions, Zourrig, et al. [85] stated that customers will tend towards seeking revenge on an active basis. By influencing and responding to direct complaint behaviors, organizations can reduce consumers’ experiences of dissatisfaction and hostility and improve the overall evaluations of their services, moderating the generation of negative word-of-mouth [86]. The majority of prior studies on trust repair have focused on commercial transaction behaviors and have rarely delved into the healthcare industry as a whole, much less those targeting orthodontics.

Using orthodontic treatments as an example, this study analyzed the effects of trust repair strategies on trust repair and the effects of trust repair on satisfaction and word-of-mouth in the event of a medical dispute. It also examined whether third-party involvement through mediation efforts can enhance the relationship between trust repair and patient satisfaction/word-of-mouth. The study also investigated whether trust repair plays a mediating role between trust repair strategies and patient satisfaction/word-of-mouth. An online survey was adopted, and a total of 353 valid responses were retrieved. The results of the analysis demonstrated that, of the compensation strategies devised by healthcare institutions for patients in the event of a medical dispute, affective and informational repair strategies have a significantly positive effect on trust repair, while functional repair strategies have no significant influence on trust repair. In terms of the moderating effect of third-party involvement, the results revealed that the path coefficients between trust repair and patient satisfaction/word-of-mouth were higher in the group with third-party involvement than in the one without, but a statistically significant difference was only achievable in the relationship between trust repair and word-of-mouth. In addition, the results of the mediation analysis showed that trust repair mediates between affective/informational repair and patient satisfaction/word-of-mouth, but there is no such mediating effect between functional repair and patient satisfaction/word-of-mouth. The above findings indicate many important academic and practical implications, as detailed below.

First, in terms of the relationship between affective, functional, and informational repair strategies and trust repair, this study established that affective and informational repair can indeed predict trust repair in patients, and that informational repair has a greater effect on trust repair than affective repair. In other words, if a medical dispute arises as a result of the patient discerning a mismatch between the expected and actual outcomes after undergoing an orthodontic treatment, the affective and informational repair undertaken by the healthcare institution can effectively restore the patient’s trust. While affective compensation allows the patient to perceive the sincerity and remorse of the healthcare institution, making good use of information to communicate with and offer tangible explanations to the patient enables them to grasp the medical issues and risks that may arise on their behalf. This finding was consistent with that of previous trust-related literature [29,87]. Nevertheless, the results of this study also showed that functional repair is unable to predict trust repair, suggesting that patients’ trust may remain effectively unrestored even when healthcare institutions attempt to make amends for a medical dispute by financial means. This result was contrary to the findings of previous studies [35,88]. A possible explanation is that, from the patients’ perspective, the medical dispute may involve an undeniable medical error that money alone cannot amend; financial compensation may deliver patients with the impression that the healthcare institutions are merely buying them off and without due attention to the medical care transgression. Consequently, the result was insignificant. In the event of a medical error, rather than receive monetary compensation, the patient usually prefers for the healthcare institution to acknowledge its fault, offer a sincere apology as a means of affective repair, and engage in active and effective communications to clarify/explain the root cause of error as a means of informational repair. On the basis of the findings, when a medical error does occur, healthcare institutions are advised to take the first opportunity to acknowledge their mistakes, demonstrate empathy, offer patients and their family members’ emotional support, and pledge their willingness to offer compensation. Meanwhile, practical efforts are strongly recommended to offer additional medical information or to seek alternative medical remedies, among other things, to allay the anxiety experienced by patients and their family members.

Second, with regard to the relationship between trust repair and patient satisfaction and word-of-mouth, trust is widely recognized as an essential antecedent of various consumer behaviors [42,89]. The results of this study showed that, in the healthcare industry, trust repair can still positively and significantly be used to predict patient satisfaction and word-of-mouth, and it has a greater effect on the former than on the latter. These findings are in line with the results of prior evidence pertaining to satisfaction and word-of-mouth [52,60,61]. In light of these findings, after a medical error has taken place, healthcare institutions are advised to propose a comprehensive set of compensation strategies, invite patients back for free follow-up appointments, and even encourage them to share information with their friends. This will allow the healthcare institutions to not only generate favorable word-of-mouth but also build a good reputation among patients and the community.

Third, the results of the moderating effect of third-party involvement on the relationship between trust repair and patient satisfaction/word-of-mouth revealed that although trust repair has a greater effect on patient satisfaction when there is third-party involvement than when there is not, the underlying difference is not statistically significant. This is inconsistent with the finding of previous research stating that consumers with higher levels of organizational trust are more likely to experience increased levels of satisfaction [51]. A possible reason is that the level of satisfaction cannot be enhanced when a medical error has already transpired, but it is possible to prevent the amplification of the negative emotions involved. Trust repair was also found to have a significantly greater effect on word-of-mouth when there is third-party involvement than when there is not, which is in line with the findings of previous literature related to word-of-mouth events [60,61]. Thus, during the process of trust repair, third parties can offer assistance to healthcare institutions while improving the outcomes of trust repair among patients. In view of the above findings, healthcare institutions are advised to maintain long-term cooperation with third-party institutions through the provision of information, case counseling, and other means in order to continuously understand patients’ needs and to promote a positive word-of-mouth healthcare environment.

Fourth, with regard to the mediating role of trust repair between the three types of trust repair strategies and patient satisfaction/word-of-mouth, the results confirmed that trust repair mediates between the relationship of affective repair and patient satisfaction/word-of-mouth, and that between informational repair and patient satisfaction/word-of-mouth. These findings resonate with the viewpoint of previous literature related to trust repair [31]. However, the same mediating effect was unobserved between functional repair and patient satisfaction/word-of-mouth. This finding contrasted with the viewpoint of previous literature associated with trust repair [31], possibly due to the limited effectiveness of functional repair in significant response to medical error necessitating intervention. In view of the above findings, healthcare institutions are advised to adopt affective and informational repair methods as their first line of response in order to produce better outcomes for patient satisfaction and word-of-mouth and to mitigate compensatory outlays. By its very nature, an orthodontic treatment is in fact a highly complex, error-prone, and time-consuming process that is difficult to skip. It also requires repeated adjustments and the devotion of a great deal of money and time by patients/relations. Considering all these inputs, any achievement regarding the goals of repairing strategies will be easier to obtain patients’ cooperation by way of trust repair should any medical dispute occur.

As the questionnaire in this study was distributed mainly in the form of an online survey to targeted online communities as the subjects of the investigation, the sample collected was limited and may not represent the opinions of the entire population. Subsequent studies may focus on non-online samples for further comparisons with the above results. Moreover, the current study drew only on the example of orthodontic treatments, when different medical treatments vary in their nature, prevalence, and attributes. Future studies can explore different medical behaviors to examine the effectiveness of different compensatory strategies and the reactions to third-party involvement leading to moderation.

## 6. Conclusions

Trust has always been a compelling interest across all industries, and it is essential for the establishment of interpersonal relationships or the transactional facilitation. This study focused on a healthcare context to analyze the potential effect of affective, functional, and informational repair strategies in response to medical disputes and the resulting mediation. Generally speaking, whenever a substantive error arises in a commercial transaction, offering consumers substantive financial compensation in the form of money yields effective results. When it comes to medically related disputes, however, the results obtained in this study were quite unusual, since patients appeared to become apathetic regarding the functional repair and compensation strategies proposed by most healthcare institutions. This study inferred that, since medical treatments are linked to patients’ physical or psychological well-being, any error that occurs tends to be irreversible, psychologically- or emotionally speaking. Losses incurred by most commercial transactions, on the other hand, are frequently offset by the offer of brand-new commodities or monetary compensation, and they will not lead to any physical and psychological loss for consumers. Therefore, in the event of a medical error, healthcare institutions can adopt affective and informational compensation strategies that convey a sense of marked sincerity and that make patients feel valued in order to effectively restore their trust and, in turn, improve their satisfaction and word-of-mouth experience.

## Figures and Tables

**Table 1:** Respondent characteristics.

Characteristics		Frequency	%
Gender	Male	118	33.43
	Female	235	66.57
Age	≤20	15	4.25
	21–30	147	41.64
	31–40	123	34.84
	41–50	57	16.15
	≥51	11	3.12
Education	High school	44	12.46
	College/University	271	76.77
	Graduate school	38	10.76
Orthodontics cost (in USD)	≤1700	85	24.08
	1700–2650	162	45.89
	2650–3970	77	21.81
	≥3970	29	8.22
Study scenario	Without third-party involvement	147	33.43
	With third-party involvement	206	66.57

**Table 2:** Reliability and validity.

Latent Variable	Items	Factor Loading	Cronbach’s Alpha	CR	AVE	Correlations					
						AR	FR	IR	TR	WOM	SAT
Affective repair (AR)	3	0.78–0.84	0.85	0.86	0.67	**0.82**					
Functional repair (FR)	3	0.50–0.86	0.72	0.74	0.50	0.41	**0.71**				
Informational repair (IR)	3	0.77–0.82	0.83	0.84	0.63	0.61	0.58	**0.79**			
Trust repair (TR)	4	0.76–0.84	0.87	0.87	0.63	0.57	0.43	0.64	**0.80**		
Word-of-mouth (WOM)	3	0.81–0.86	0.87	0.88	0.70	0.49	0.35	0.51	0.66	**0.84**	
Satisfaction (SAT)	3	0.80–0.84	0.86	0.86	0.67	0.56	0.50	0.66	0.68	0.70	**0.82**

*Note*: CR = composite reliability; AVE = average variance extraction; diagonal boldfaces indicate the square root of AVE.

**Table 3:** Heterotrait–monotrait ratio of correlations.

	AR	FR	IR	TR	WOM	SAT
Affective repair (AR)						
Functional repair (FR)	0.48					
Informational repair (IR)	0.72	0.74				
Trust repair (TR)	0.66	0.53	0.74			
Word-of-mouth (WOM)	0.56	0.44	0.60	0.75		
Satisfaction (SAT)	0.65	0.63	0.78	0.79	0.80	

**Table 4:** Hypothesis testing results.

Hypothesis	Path	β	*z* Value	*p* Value	Support?
H1-a	Affective repair→Trust repair	0.27	3.66	<0.001	Yes
H1-b	Functional repair→Trust repair	0.00	−0.01	0.989	No
H1-c	Informational repair→Trust repair	0.59	5.44	<0.001	Yes
H2-a	Trust repair→Satisfaction	0.82	13.41	<0.001	Yes
H2-b	Trust repair→Word-of-mouth	0.76	12.26	<0.001	Yes

**Table 5:** Model fit.

Index	Measurement Model	Structural Model	Threshold	References
χ^2^/df	1.94	2.21	<3	Hartwick and Barki [81]
GFI	0.95	0.95	≥0.9	Gefen, Straub and Boudreau [80]
RMSEA	0.05	0.06	<0.08	Gefen, Straub and Boudreau [80]
SRMR	0.03	0.05	<0.1	Hair, Black, Babin and Anderson [77]
CFI	0.97	0.97	≥0.9	Hartwick and Barki [81]
NFI	0.94	0.94	≥0.9	Gefen, Straub and Boudreau [80]
AGFI	0.93	0.93	≥0.8	Gefen, Straub and Boudreau [80]
PNFI	0.75	0.75	≥0.5	Wu, Yang and Koo [82]

*Note*: χ^2^/df = ratio between Chi-square and degrees of freedom; GFI = goodness of fit index; RMSEA = root mean square error of approximation; SRMR = standardized root mean square residual; CFI = comparative fit index; NFI = normed fit index; AGFI = adjusted goodness of fit index; PNFI = parsimony normed fit index.

**Table 6:** Moderating effect testing results.

Path	Scenario	β	SE	Wald Test	
				χ^2^	*p* Value
Trust repair→Satisfaction	Without third party (*n* = 147)	0.76	0.06	0.03	0.854
Trust repair→Satisfaction	With third party (*n* = 206)	0.86	0.04		
Trust repair→Word-of-mouth	Without third party (*n* = 147)	0.67	0.08	5.49	0.019
Trust repair→Word-of-mouth	With third party (*n* = 206)	0.81	0.04		

*Note*: β is the path coefficient; SE = standard error.

**Table 7:** Mediation analysis.

Path	Direct Effect	Indirect Effect	Standard Error	95% CI		Type of Mediation
Affective repair→Satisfaction	0.110		0.042	0.027	0.193	
Functional repair→Satisfaction	0.146		0.049	0.050	0.241	
Informational repair→Satisfaction	0.289		0.059	0.174	0.405	
Affective repair→Trust repair→Satisfaction		0.100	0.021	0.066	0.137	Partial mediation
Functional repair→Trust repair→Satisfaction		0.010	0.009	−0.002	0.026	No mediation
Informational repair→Trust repair→Satisfaction		0.125	0.028	0.081	0.172	Partial mediation
Affective repair→Word-of-mouth	0.130		0.053	0.027	0.233	
Functional repair→Word-of-mouth	0.019		0.061	−0.100	0.139	
Informational repair→Word-of-mouth	0.120		0.073	−0.024	0.264	
Affective repair→Trust repair→Word-of-mouth		0.146	0.030	0.099	0.197	Partial mediation
Functional repair→Trust repair→Word-of-mouth		0.001	0.006	−0.007	0.011	No mediation
Informational repair→Trust repair→Word-of-mouth		0.052	0.029	0.006	0.100	Full mediation

*Note*: CI = confidence interval.

## Data Availability

The data that support the findings of this study are available from the corresponding author upon reasonable request.

## Appendix A. Questionnaire Items

**Table:**

**Constructs**	**Items**	**Sources**
Affective repair	I think the healthcare institution has made an obvious apology.	Xie and Peng [28]
	I think that I received affective compensation (e.g., apologize in person).	
	I think the healthcare institution has taken patients’ emotions into account in responding to the negative publicity.	
Functional repair	I received concrete compensation.	Xie and Peng [28]
	I think the healthcare institution has made functional efforts (e.g., money back, discount) in response to the negative publicity.	
	I think the healthcare institution has made economic compensation for losses in negative publicity.	
Information restoration	The healthcare institution has responded to this incident.	Xie and Peng [28]
	The healthcare institution’s response contains the necessary information.	
	The healthcare institution has provided me with the information I need about this incident.	
Trust repair	I am willing for my trust to be repaired.	Sedikides [68]
	I think the trust repair can help to reduce negative emotions toward the incident.	
	I think the trust repair has restored my trust.	
	I think the trust repair has given me confidence.	
Word-of-mouth	I will say positive things about this healthcare institution to other people.	Babin, Lee, Kim and Griffin [72]
	I will recommend this healthcare institution to someone who seeks my advice.	
	I will encourage friends and relatives to visit this healthcare institution.	
Satisfaction	Overall, I think this healthcare institution has proposed a satisfactory solution to the mistake.	Gelbrich, Gäthke and Grégoire [69], Goodwin and Ross [70], Holloway, Wang and Parish [71]
	Overall, I was satisfied with the way this healthcare institution dealt with the errors.	
	I was happy with this patient experience.	

## Appendix B. Study Scenarios

**Scenario 1:**

In response to an error in its medical services, the healthcare institution in question offers to adopt the following compensation strategies: (1) expressing care for your welfare, apologizing, and showing remorse; (2) offering monetary recompense, discounts, and premium upgrades; and (3) explaining the error and communicating fully with you regarding remedy. Please answer the questionnaire with this scenario in mind.

**Scenario 2:**

In response to an error in its medical services, the healthcare institution in question offers to adopt the following compensation strategies: (1) expressing care for your welfare, apologizing, and showing remorse; (2) offering monetary recompense, discounts, and premium upgrades; and (3) explaining the error by communicating fully with you with the involvement of an impartial third party (e.g., the local health bureau, a physicians’ association, the Taiwan Healthcare Reform Foundation, or the Taiwan Dental Association) to mediate in the dispute. The purpose of the mediation will be to help narrow any cognitive gap between you and the respective healthcare institution, resolve your dispute, and facilitate reconciliation. Please answer the questionnaire with this scenario in mind.
